# Psychometric Evaluation of the Core Competencies in Disaster Nursing Scale for Disaster Rescue Nurses in Mainland China

**DOI:** 10.1155/jonm/9957270

**Published:** 2025-08-10

**Authors:** Jinjia Lai, Hongyi Chen, Yongqi Huang, Chaoqun Ma, Ping Zeng, Hanxi Chen, Yibing Tan

**Affiliations:** ^1^Emergency Department, The First Affiliated Hospital of Guangzhou University of Chinese Medicine, Guangzhou, Guangdong, China; ^2^School of Nursing, Guangzhou University of Chinese Medicine, Guangzhou, Guangdong, China; ^3^Department of Nursing, Guangzhou Red Cross Hospital (Guangzhou Red Cross Hospital of Jinan University), Guangzhou, Guangdong, China

**Keywords:** core competencies, disaster nursing, disaster rescue nurse, psychometric test, reliability validity

## Abstract

**Background:** Disaster rescue nurses (DRNs) play an essential role in disaster medical rescue. To ensure an effective response, it is imperative to assess the core competencies of DRNs. In 2019, the International Council of Nurses (ICNs) published the Core Competencies in Disaster Nursing Version 2.0 (CCDN V2.0), which outlines 33 competencies for DRNs. However, these competencies currently lack psychometric evaluation in China, which limits the direct assessment of DRNs' core competencies in disaster nursing and the effectiveness of disaster nursing education.

**Objectives:** The objective of this study was to develop and validate the Core Competencies in Disaster Nursing Scale for Disaster Rescue Nurses (CCDNS-DRNs).

**Design:** A methodological and cross-sectional design was used to develop and validate this scale.

**Methods:** The researchers developed an initial scale using cross-cultural translation. Expert consultation was utilized to validate the content validity. A total of 401 nurses were surveyed by purposive sampling. They completed a general information questionnaire along with the CCDNS-DRN. Data analysis was performed using SPSS 26.0, Mplus 8.3, and R 4.5.0 and included general statistical analysis, classical test theory (CTT), and item response theory (IRT) analyses.

**Results:** A 33-item eight-factor CCDNS-DRN was developed with satisfactory validity and reliability, with a content validity index of 0.980 for the total scale and a content validity index of 0.780–1.00 for each item. The confirmatory factor analysis showed that *x*^2^*/df*: 2.940, CFI: 0.933, TLI: 0.923, RMSEA: 0.069, and SRMR: 0.036. The Cronbach's alpha, split-half validity, and internal correlation coefficient (ICC) of the scale were 0.978, 0.918, and 0.898, respectively. The results of the IRT revealed that the discrimination indices of all items ranged from 2.037 to 4.970, the difficulty parameters of all items ranged from −2.837 to 1.396, and the average information values for the items ranged from 0.961 to 3.424.

**Conclusions:** The CCDNS-DRN exhibits good psychometric properties, providing a scientifically sound and effective instrument to evaluate the core competencies in disaster nursing among DRNs in China.

## 1. Introduction

According to the Global Disaster Data Platform (GDDP), 957 disaster events occurred globally in 2023, affecting approximately 200 million people [1]. These disasters pose significant threats to human health [2, 3]. As the largest group within the healthcare system, nurses play an indispensable role and emerge as a crucial force in safeguarding public health [4–6].

Disaster rescue nurses (DRNs) refer to those individuals who have undergone systematic education and training in disaster nursing, thereby becoming specialized nurses equipped to respond to disasters and emergencies [7]. During the disaster rescue, DRNs leverage their extensive knowledge, skills, and expertise to promptly adapt to complex rescue environments, enabling them to perform tasks beyond the capabilities of general nurses [8, 9]. Consequently, the core competencies in disaster nursing of DRNs directly impact the quality and efficiency of medical disaster relief operations [10].

Disaster nursing education in China started relatively late, and the training of DRNs is still in its early stages [11, 12]. Assessing the core competencies in disaster nursing of disaster relief nurses will contribute to the construction of the education and training system. At present, tools for assessing core competencies in disaster nursing are mostly designed for nursing students or general nurses [13, 14]. Given the varied roles that nurses assume in emergency medical rescue [15], it is not appropriate to apply a uniform standard to measure their competency in disaster nursing. Therefore, it is necessary to develop a scale to measure core competencies in disaster nursing of DRNs, thereby offering an effective tool to continuously monitor and improve the quality of disaster nursing education for DRNs.

## 2. Background

In recent years, affected by the frequent occurrence of disaster events, China has attached great importance to the construction of Emergency Medical Team (EMT) [16]. By the end of 2022, China had successfully established 59 national-level EMTs, among which EMTs from Shanghai, Guangdong, Tianjin, Sichuan, and Macao had obtained the WHO certification assessment [17]. In addition, provinces were mandated to establish additional teams at the provincial, municipal, and county levels, culminating in a total of 6500 teams nationwide [18]). EMTs are multifaceted teams that consist of various medical professionals, with nurses typically occupying a pivotal role. Recognizing this, several provinces in China have begun to explore the educational programs of disaster nursing specialist nurses. For instance, the Guangdong Nursing Association has been organizing annual continuing education programs for disaster nursing specialist nurses since 2019, leading to the training of around 200 provincial-level disaster nursing specialists [19]. Both EMT nurses and disaster nursing specialist nurses constitute an integral part of the DRNs in China, underlining their significance in emergency response efforts.

In 2019, the International Council of Nurses (ICNs) released the Core Competencies in Disaster Nursing Version 2.0 (CCDN V2.0) [20], a comprehensive framework outlining eight essential domains for disaster nursing: preparation and planning, communication, incident management, safety and security, assessment, intervention, recovery, and law and ethics [20]. This framework stratifies disaster nursing competencies into three levels based on the varying roles and responsibilities of nurses. At the II Level, nurses are defined as any nurse who has achieved the Level I competencies and is/aspires to be a designated disaster responder within an institution, organization, or system. DRNs typically have undergone structured disaster nursing education and are officially designated as disaster responders in their institutions [19, 21], closely aligning with the characteristics of Level II nurses as defined in the CCDN V2.0. The CCDN V2.0 serves as a benchmark, establishing a standard that is often higher than the current practical level of disaster nursing education. This standard represents an aspirational goal that guides the continuous improvement and advancement of disaster nursing education worldwide [11].

Given the nascent stage of disaster nursing education in China and the absence of a unified standard for training DRNs [8, 22], the CCDN V2.0 clearly articulated the 33 core competencies that DRNs need to possess and serves as a pivotal guide for the development of targeted educational programs. It is essential to assess learning outcomes in a training program [23]. However, the current instruments employed for assessing core competencies in disaster care among DRNs are inadequate, as these tools were not specifically developed for this target population [24–26]. Consequently, they may not entirely correspond with the most recent. This emphasizes the pressing need to adapt the Level II nurse competency requirements outlined in CCDN V2.0 into a reliable and valid assessment scale specifically designed for DRNs. Therefore, the objective of this study was to transform the Level II nurse competency requirements outlined in CCDN V2.0 into the Core Competencies in Disaster Nursing Scale for Disaster Response Nurses (CCDNS-DRNs) and to examine its psychometric properties using both classical test theory (CTT) and item response theory (IRT). The aim was to provide a reliable foundation for disaster nursing education and training for DRNs in China.

## 3. Methods

### 3.1. Aims

The aim of this study was to transform the Level II nurse competency requirements in CCDN V2.0 into the CCDNS-DRN and test its psychometric properties.

### 3.2. The Scale and Its Development Phases

**Figure 1:**
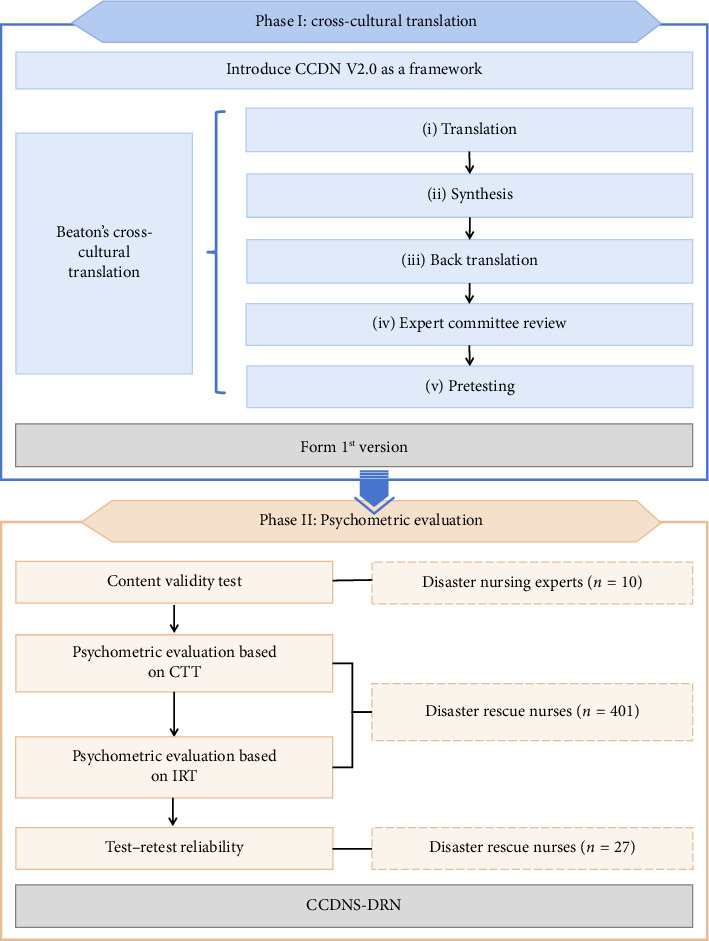
The development process of the CCDNS-DRN.

The CCDNS-DRN was developed in two phases: (I) cross-cultural translation and (II) psychometric evaluation (Figure 1).

#### 3.2.1. Phase I: Cross-Cultural Translation

In June 2022, we communicated the purpose of this study to the ICN via email and obtained their authorization. In June 2023, the Level II nurse competency requirements in CCDN V2.0 (8 domains, 33 items) were translated into Chinese following Beaton's cross-cultural translation process [27]. The process includes (i) translation, (ii) synthesis, (iii) back translation, (iv) expert committee review, and (v) pretesting.

During the translation process, we engaged two accomplished scholars as translators to render the CCDN V2.0 Level II nurse competency requirements into Chinese. One translator was a seasoned college English instructor with extensive experience in English pedagogy, while the other was an emergency nursing specialist who had served as a member of the Chinese medical mission in Ghana, providing valuable domain expertise. In the synthesis phase, the two translators collaborated closely with us, meticulously reviewing the translated manuscript to reach a consensus and produce a coherent, unified document. To ensure the accuracy and fidelity of the translation, we subsequently employed a back-translation method. Two independent scholars, unfamiliar with CCDN V2.0, were invited to participate. One scholar held a Master's degree in nursing and had international work experience, while the other was a college English teacher without a medical background, thereby providing diverse perspectives for the translation.

To further validate the translated manuscript, we assembled a panel of 10 disaster nursing experts to collectively review both the translated document and the original CCDN V2.0, ensuring the translation's professionalism and technical accuracy. Following this rigorous review process, we conducted cognitive interviews with 18 DRNs to assess their comprehension of the translated scale. The primary aim of these interviews was to uncover any potential issues or challenges that might arise during the scale's practical application, thereby enhancing the reliability and validity of its measurement outcomes.

#### 3.2.2. Phase II: Psychometric Evaluation

##### 3.2.2.1. Step 1: Expert Consultation

It is recommended that a scale should be sent to at least seven experts to assess its suitability for language and content [28]. Therefore, we invited experts (*N* = 10) from the Disaster Nursing Specialized Committee of the Chinese Nursing Association to evaluate the content validity of this scale. The experts were asked to assess the relevance and clarity of the items using a four-point rating method [29]. Based on the experts' ratings, the content validity indices of the scale were calculated separately for relevance and clarity, including Item-Content Validity Index (I-CVI) and the scale-level content validity indices (S-CVIs). The S-CVI used two methods: S-CVI Average (S-CVI/Ave) and the S-CVI Universal Agreement (S-CVI/UA) among experts. A value of I-CVI above 0.78, S-CVI/UA above 0.80, and S-CVI/Ave above 0.90 indicated excellent content validity [30, 31].

##### 3.2.2.2. Step 2: Psychometric Evaluation Based on CTT

The confirmatory factor analysis (CFA) was used to test the factor structure of the scale [32]. The model construction was conducted using Mplus Version 8.3 software. Model fit was evaluated using a comprehensive set of indices, including the chi-square/degree of freedom (*x*^2^*/df*), root mean square error of approximation (RMSEA), standardized root mean square residual (SRMR), comparative fit index (CFI), and Tucker–Lewis index (TLI) [33]. A value of *x*^2^*/df* below 3, RMSEA below 0.08, SRMR below 0.08, CFI above 0.9, and TLI above 0.9 indicated excellent model fit [34, 35]. Furthermore, grounded in the theoretical foundations of the model, any refinements to the model structure were guided by the Modification Index (MI).

Cronbach's α was used to test the internal reliability of the scale. When the value of Cronbach's α exceeds 0.70, it indicates acceptable internal consistency reliability of the scale, and values above 0.80 denote excellent internal consistency reliability [36]. The internal correlation coefficient (ICC) was used to evaluate the test–retest reliability of the scale. A value of ICC above 0.7 indicates that the scale has good stability [37].

##### 3.2.2.3. Step 3: Psychometric Evaluation Based on CTT

This study conducted IRT analysis of the scale using the multidimensional item response theory (MIRT) package in R 4.5.0 such that each item is described by discrimination parameter (*a*), threshold parameters (*b*_1_, *b*_2_, *b*_3_, and *b*_4_), and average information [38]. (i) Discrimination parameter (*a*): reflects an item's ability to differentiate between participants with varying trait levels. Higher *a* values indicate stronger discriminative power, with *a* > 0.6 considered acceptable. (ii) Threshold parameters correspond to the *θ* level of the latent trait necessary to respond with the corresponding anchor. When the value range of *b* is between −4 and 4, it indicates that the difficulty of the item is moderate and the option classification is reasonable. (iii) The average information is the average value of the item information function at five characteristic levels (*θ* = −2, −1, 0, 1, and 2). When the average information amount is 25, the test quality evaluation is better (equivalent to measurement error = 0.2). This scale consisted of 33 items; the value of average information > 0.756 (25/33 items) indicates excellent measurement precision.

### 3.3. Participants

In November 2023, we established contact with the nursing managers of the emergency rescue teams and the heads of the Guangdong Provincial Disaster Care Professional Committee. After obtaining their consent, we distributed electronic questionnaires to the target population. The inclusion criteria for participants are as follows: (1) obtained the national nurse qualification certificate, (2) received systematic disaster care education during the work, (3) obtained the provincial-level or above certificate of disaster nursing specialist nurse or been a member of the provincial-level or above EMT, and (4) informed consent and voluntary participation in this study. Exclusion criteria are as follows: (1) no longer engaged in clinical nursing work and (2) those who are not on duty due to further studies, maternity leave, etc. Removal criteria for the samples are as follows: (1) samples with regular answers and (2) samples with a total answering time of below 2 minutes.

### 3.4. Data Collection

From November 2023 to December 2024, using the purposive sampling method, online questionnaires were distributed to DRNs in Guangdong, Sichuan, Chongqing, and other provinces via the WJX app, a widely used online survey platform in China. The survey content included (1) general information questionnaire: gender, age, highest education, hospital level, years of work, professional title, work department, and disaster nursing background and (2) the CCDNS-DRN, which consists of 33 items with eight domains: preparedness and planning (5 items), communication (4 items), incident management (4 items), safety and security (5 items), assessment (4 items), intervention (6 items), recovery (2 items), and law and ethics (3 items). Each item was scored on a 5-point Likert scale (1 = *strongly disagree*, 2 = *disagree*, 3 = *uncertain*, 4 = *agree*, and 5 = *strongly agree*), with higher scores implying higher competence. During the survey, the contact information of 27 participants was retained, and they were surveyed again two weeks later to assess the scale's test–retest reliability. Participants were required to complete all the questions before submitting the electronic questionnaire, ensuring no incomplete submissions. To prevent duplicate entries, the WJX app was configured to allow each user to participate in the survey only once.

### 3.5. Ethical Considerations

Before the study, a description was provided on the homepage of the WJX app. All participants were informed and voluntarily agreed to participate in the survey, with their information kept strictly confidential. This study received a priori ethical approval by the institutional review board of the study site (no. YE202223801).

## 4. Results

The results were presented according to the CCDNS-DRN's development process: (I) cross-cultural translation and (II) psychometric evaluation.

### 4.1. Phase I: Cross-Cultural Translation

According to the Beaton's cross-cultural translation process, we translated the Level II nurse competency requirements in CCDN V2.0 into Chinese. During the expert committee review, 10 disaster nursing experts were invited to thoroughly discuss the original document, translation manuscript, and back-translation manuscript. Among the 10 experts, 4 held a doctoral degree, 5 held a master's degree, and 1 held a bachelor's degree. The average age of the experts was 44.60 ± 7.06 years old; 7 experts held senior professional titles and 3 experts held associate senior professional titles, with an average working time of 23.90 ± 8.33 years. The 10 experts provided 22 modification suggestions for the items, providing critical intellectual contributions to the cross-cultural translation.

In the pretesting phase, this study conducted three rounds of cognitive interviews (Table 1). In the first round, 8 DRNs were interviewed, and they raised questions about 22 items. The results showed that most of the problems with the items were “semantically ambiguous” and “difficulty in understanding.” After the discussion at the expert meeting, the relevant items were revised. For example, in Item 1, the term “emergency drills” has been revised to “disaster emergency drills,” and in Item 5, the term “refresher course” has been revised to “continuing education.” In the second round, 5 DRNs were interviewed, and they raised questions about 6 items. The main adjustments include “language redundancy” and “item word order” and other issues. Given that the questions were generally well comprehended, we revised these items through group discussions. In the third round, 5 DRNs were interviewed, and all the interviewees indicated that they had not any doubts about the semantic equivalence, connotation understanding, and cultural expression of all items. Consequently, this stage produced the second version of the scale. Ultimately, the first version of the scale was established through cross-cultural translation.

### 4.2. Phase II: Psychometric Evaluation

#### 4.2.1. Basic Characteristics of the Participants

A total of 401 participants were included in this phase. Most of the participants were female (76.81%), the average age was (35.64 ± 5.61) years old, the average nursing experience was (13.62 ± 6.51) years, and other characteristics of the participants are shown in Table 2.

#### 4.2.2. Step 1: Content Validity Test

The experts' consultation results showed that the score range of I-CVI of this scale was 0.78–1.0, S-CVI/UA was 0.85, and S-CVI/Ave was 0.98.

#### 4.2.3. Step 2: Psychometric Evaluation Based on CTT

**Figure 2:**
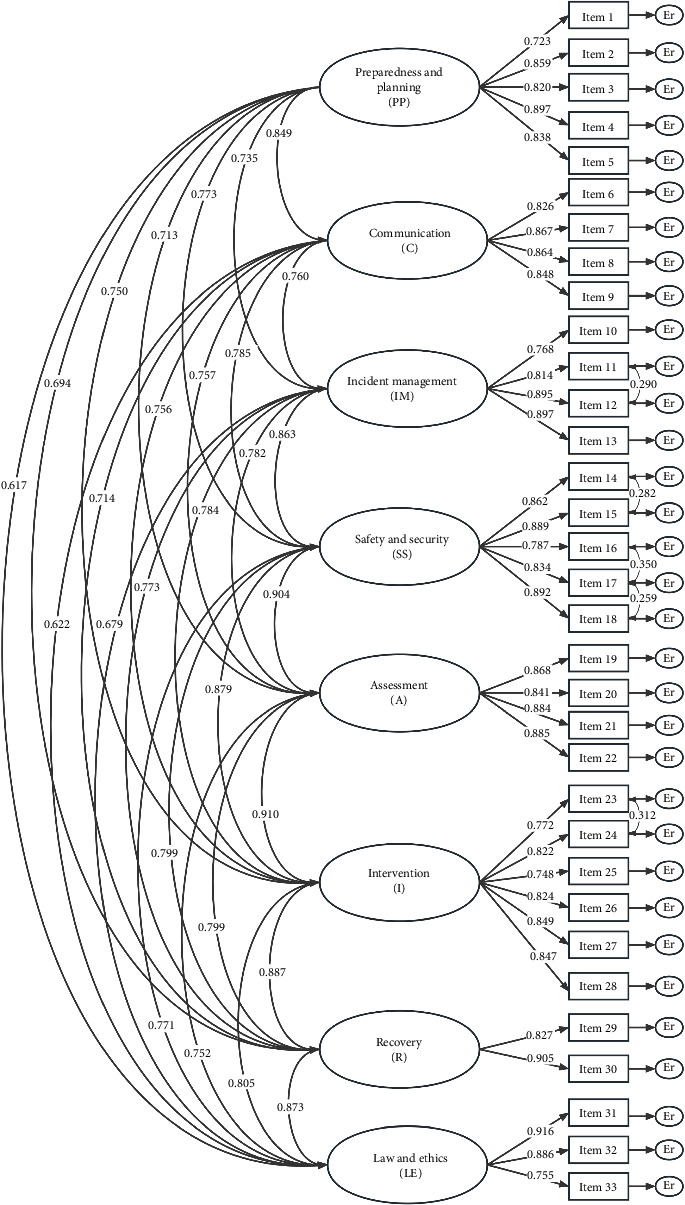
The CFA results of the 8-factor model of CCDNS-DRN.

According to the original framework of CCDN V2.0, the eight domains were used as latent variables: (i) preparation and planning, (ii) communication, (iii) incident management, (iv) safety and security, (v) assessment, (vi) intervention, (vii) recovery, and (viii) law and ethics. The factor structure of 33 items was used as the manifest variable to establish the CFA model. The results showed that the initial model fit was mediocre (*x*^2^*/df*: 3.253, CFI: 0.921, TLI: 0.910, RMSEA: 0.075, and SRMR: 0.039). To this end, we added path coefficients between Item 11 and Item 12, Item 14 and Item 15, Item 15 and Item 16, Item 16 and Item 17, and Item 23 and Item 24 according to the recommendations of the MI. The results showed that the fit indices of the modified model were all improved (*x*^2^*/df*: 2.940, CFI: 0.933, TLI: 0.923, RMSEA: 0.069, and SRMR: 0.036). The standardized factor loadings of all items in the modified model ranged from 0.723 to 0.916, all exceeding 0.7, indicating that the scale demonstrated good construct validity (Figure 2).

The reliability of this scale was tested using Cronbach's α coefficient and split-half reliability. The results showed that Cronbach's α of the total scale was 0.978, and Cronbach's α of each domain ranged from 0.853 to 0.938. This demonstrated that the scale exhibited adequate reliability. The split-half reliability of the total scale was 0.918 and that of each domain ranged from 0.704 to 0.907, indicating good internal consistency across the scale and its subdomains. The second measurement was conducted on 27 participants after two weeks. The results showed that ICC of the total scale was 0.898, and the ICC of each domain ranged from 0.727 to 0.860, revealing that this scale had good stability across time (Table 3).

#### 4.2.4. Step 3: Psychometric Evaluation Based on CTT

Both the graded response model (GRM) and the generalized partial credit model (GPCM) are suitable for analyzing polytomous datasets. Compared with GPCM, the AIC and BIC of GRM were smaller, and the value of log-likelihood (logLik) was larger, indicating that the fitting effect of the GRM was better (Table 4). Therefore, GRM is used for IRT in this study. The results revealed that the discrimination indices of all items ranged from 2.037 to 4.970, with all values exceeding 0.6, indicating that all items demonstrate good discrimination. The difficulty parameters of all items ranged from −2.837 to 1.396, showing a unidirectional increasing trend aligned with ascending difficulty levels (*b*_1_–*b*_4_) without any reversed thresholds, thereby confirming compliance with established requirements. The average information of the items was distributed between 0.961 and 3.424, all surpassing the benchmark value of 0.756 (25/33). The discrimination indices, difficulty parameters, and average information quantities of each item are presented in Table 5, while the item characteristic curves are illustrated in Supporting file 1.

## 5. Discussion

This study aimed to develop the CCDNS-DRN under the guidance of CCDN V2.0. We combined CTT and IRT psychometric strategies to select items and examine the reliability and validity of the CCDNS-DRN. The final version includes 33 items across eight domains, which comprehensively reflect the core competency elements recommended by the ICN for DRNs in disaster nursing. The scale can serve as a basis for developing a systematic, independent, and context-specific disaster nursing education program for DRNs in China.

In cross-cultural translation, we adopted Beaton's cross-cultural translation process [27]. The process includes translation, synthesis, back translation, expert committee review, and pretesting and is widely recognized for its clarity and simplicity [39–41]. The scholars involved in the translation and back translation had solid expertise and extensive professional experience, ensuring content validity as well as semantic, technical, criterion, and conceptual equivalence between the translated Chinese version and the original document [42, 43]. Researchers developing scales often overestimate respondents' understanding of questionnaire items, leading to response errors [44]. To address this issue, we conducted three rounds of cognitive interviews during the pretesting, engaging in verbal interactions with the scale's target groups, and identified 28 potential issues that the scale might encounter in practical use, thereby enhancing the scientific rigor of the scale's development.

Psychometric analysis is a crucial phase in scale development. In the context of CTT, I-CVI and S-CVI of the CCDNS-DRN exceeded the recommended thresholds, indicating strong content validity. It is worth noting that when issuing the CCDN V2.0, the ICN had clearly outlined the hierarchical relationship between each core competency and domain. To ensure consistency with the CCDN V2.0 guidelines, we directly employed the CFA to explore the factor structure of our scale, following a thorough literature review and conference discussions. The results demonstrated that all fit indices of the model fell within acceptable ranges, thereby ensuring the alignment of our scale with the CCDN V2.0. Reliability is an important metric of the stability and consistency of a scale's measurements [37]. In this study, Cronbach's *α*, split-half reliability, and test–retest reliability were performed to test the reliability of CCDNS-DRN. The results showed that the Cronbach's α of this scale was 0.978, the split-half reliability was 0.918, and the test–retest reliability was 0.898, which indicates that this scale has adequate reliability and stability [45, 46].

IRT is a response function that incorporates both item and subject parameters. It serves as an indicator that allows for a comprehensive analysis of the relationship between item parameters and subject abilities [47]. By employing IRT, we were able to provide detailed information such as the degree of discrimination, item difficulty, and test function information within the CCDNS-DRN, thereby expanding upon traditional CTT methods [48]. Our data indicated that the CCDNS-DRN demonstrated high discrimination. In the safety and security subscale, providing materials to support nursing decisions and indicators for developing safety action plans often become powerful indicators for distinguishing higher from lower core competencies in disaster nursing. In the assessment subscale, the ability indicator to incorporate disaster triage principles into education showed strong discrimination in evaluating DRNs' assessment capabilities. In addition, the four difficulty parameters for each item in the scale exhibited a monotonically increasing trend with no reversed thresholds, indicating that the scoring levels were appropriately calibrated and that the overall difficulty of the scale was acceptable. The average information quantities for each item were within the set range, revealing that each item in the scale significantly contributed to estimating the level of DRNs' core competencies in disaster nursing and could provide a more accurate assessment.

The CCDNS-DRN consists of eight domains. Domain 1 (preparation and planning) includes five items to assess DRNs' ability to plan and improve emergency drills, clearly communicate roles and responsibilities, and organize disaster nursing capacity training. Domain 2 (communication) includes five items to assess DRNs' emergency communications, emergency communication, media information, and document preservation capabilities. Domain 3 (disaster management) includes three items, focusing on assessing DRNs' ability to build emergency plans, conduct postevent evaluation and improvement, and dynamically coordinate command in disaster or emergency situations. Domain 4 (safety and security) includes five items to assess the ability of resource management, infection control, psychological management, and safety risk control. Domain 5 (assessment) includes four items to assess DRNs' ability to capture emergency information, disaster triage training, develop physical and mental assessment guidelines, and identify and protect vulnerable populations. Domain 6 (intervention) includes six items, focusing on assessing DRNs' ability to develop isolation guidelines, hazardous materials disposal, volunteer resource development, and nurse deployment. Domain 7 (recovery) includes two items, focusing on assessing DRNs' ability to communicate the needs of nurses' postdisaster recovery responsibilities to leaders and to update and revise medical referral lists. Domain 8 (recovery) includes three items, focusing on assessing DRNs' ability to formulate emergency rescue policies and resource allocation frameworks.

### 5.1. Limitations

This study has some limitations. First, this study translated CCDN V2.0 into Chinese, and the sample mainly originated from southern China. Consequently, it may reduce the representativeness of the sample and limit the generalizability of the scale. In the future, the CCDN V2.0 can be translated and transformed into scales in different languages to support disaster nursing education programs across different countries. Second, it is recognized that a multitude of disaster types exists, and this scale can be used to measure the comprehensive competence in disaster nursing of DRNs rather than the competence in a specific disaster. Therefore, by the guidance of CCDN V2.0, specialized disaster nursing competence scales could be developed for particular disaster types in future research.

## 6. Conclusions

Guided by CCDN V2.0, this study developed the CCDNS-DRN, a 33-item, eight-factor scale scored on a 5-point Likert scale. The scale provided a measurement tool for comprehensively assessing the core competencies in disaster nursing of DRNs, and it exhibited strong psychometric properties. On the one hand, this scale will provide guidance for DRNs in clarifying their roles during disaster relief. On the other hand, it will assist nursing managers in understanding the current competence levels of DRNs in disaster nursing, thereby optimizing their disaster nursing education.

## Figures and Tables

**Table 1:** Characteristics of the participants of the cognitive interview.

Variables	The first round [*N* = 8, *n* (%)]	The second round [*N* = 5, *n* (%)]	The third round [*N* = 5, *n* (%)]
Gender			
Male	6 (25.0)	3 (60.0)	3 (60.0)
Female	2 (25.0)	2 (40.0)	2 (40.0)
Age (in years)			
≤ 35	4 (50.0)	—	2 (40.0)
> 35	4 (50.0)	5 (100)	3 (60.0)
Education			
Below bachelor's degree	1 (12.5)	—	—
Bachelor's degree or above	7 (87.5)	5 (100)	5 (100)
Years of work (years)			
5–10	1 (12.5)	—	—
10–15	6 (75.0)	3 (60.0)	2 (40.0)
≥ 15	1 (12.5)	2 (40.0)	3 (60.0)
Professional title			
Nurse in charge	8 (100)	3 (60.0)	2 (40.0)
Assistant director nurse	—	2 (40.0)	3 (60.0)
Department			
Emergency department	8 (100)	3 (60.0)	3 (60.0)
Nursing department	—	2 (40.0)	2 (40.0)
The background in disaster nursing			
Disaster specialist nurses	7 (87.5)	3 (60.0)	3 (60.0)
National EMT backbones	1 (12.5)	2 (40.0)	2 (40.0)

**Table 2:** Characteristics of the participants (*N* = 401).

Variable	*n* (%)
Gender	
Male	93 (23.19)
Female	308 (76.81)
Age (years)	
26–30	74 (18.45)
31–35	137 (34.16)
36–40	125 (31.17)
≥ 41	65 (16.21)
Education level	
College degree and below	16 (3.99)
Bachelor's degree	362 (90.27)
Master's degree and above	23 (5.74)
Hospital level	
Grade A tertiary hospital	337 (84.04)
Nongrade A tertiary hospital	64 (15.96)
Professional titles	
Nurse	8 (2.00)
Senior nurse	86 (21.45)
Nurse-in-charge	235 (58.60)
Assistant director nurse or above	72 (17.96)
Years of work (years)	
5–10	138 (34.41)
11–15	133 (33.17)
≥ 16	130 (32.42)
Clinical teachers	
Yes	317 (79.05)
No	84 (20.95)
The background of disaster nursing	
Disaster nursing specialist nurses	62 (15.46)
EMT nurses	339 (84.54)
Department	
Internal medicine	80 (19.95)
Surgery	78 (19.45)
Gynecology	4 (1.00)
Pediatrics	2 (0.50)
Emergency department	126 (31.42)
Operating room	28 (6.98)
ICU	24 (5.99)
Nursing department	7 (1.75)
Others	52 (12.97)

**Table 3:** Internal consistency reliability and test–retest reliability of the scale and each dimension.

Domains	Number of items	Cronbach's α	Split-half reliability	ICC
Preparedness and planning	5	0.907	0.866	0.727
Communication	4	0.912	0.898	0.776
Incident management	4	0.911	0.907	0.730
Safety and security	5	0.938	0.889	0.812
Assessment	4	0.925	0.904	0.847
Intervention	6	0.921	0.902	0.840
Recovery	2	0.853	0.853	0.795
Law and ethics	3	0.884	0.704	0.860
Total scale	33	0.978	0.918	0.898

**Table 4:** The model fitting results.

Model	AIC	BIC	logLik
GRM	21232.99	21891.99	−10451.49
GPCM	21522.92	22181.92	−10596.46

**Table 5:** The discrimination indices, difficulty parameters, and average information quantities of each item are presented.

Item	Discrimination *a*	Threshold				Avg information
		*b*_1_	*b*_2_	*b*_3_	*b*_4_	
Item 1	2.037	−2.592	−1.793	−0.480	0.855	0.961
Item 2	3.144	−2.635	−1.477	−0.452	0.938	1.799
Item 3	2.795	−2.837	−2.059	−0.783	0.941	1.496
Item 4	3.569	−2.189	−1.750	−0.673	0.822	2.213
Item 5	2.953	−2.281	−1.607	−0.459	1.156	1.754
Item 6	2.643	−2.410	−1.555	−0.284	1.303	1.516
Item 7	3.324	−2.473	−1.686	−0.624	1.136	1.977
Item 8	3.088	−2.420	−1.515	−0.236	1.277	1.880
Item 9	3.074	−2.212	−1.548	−0.329	1.049	1.899
Item 10	2.578	−2.162	−1.216	−0.214	1.395	1.537
Item 11	3.179	−2.468	−1.629	−0.667	1.082	1.869
Item 12	3.622	−2.218	−1.460	−0.454	1.084	2.359
Item 13	3.920	−2.358	−1.438	−0.555	0.951	2.476
Item 14	4.762	−2.253	−1.402	−0.376	0.997	3.364
Item 15	4.314	−2.285	−1.599	−0.496	0.922	2.802
Item 16	3.073	−2.605	−2.044	−0.808	0.908	1.732
Item 17	3.573	−2.387	−1.829	−0.680	0.854	2.190
Item 18	4.827	−2.256	−1.616	−0.544	0.919	3.205
Item 19	3.998	−2.243	−1.688	−0.553	1.078	2.598
Item 20	3.292	−2.289	−1.626	−0.431	1.204	2.018
Item 21	4.870	−1.841	−1.423	−0.434	0.955	3.424
Item 22	3.847	−2.040	−1.603	−0.491	1.063	2.525
Item 23	3.164	−2.291	−1.648	−0.616	1.066	1.905
Item 24	3.761	−2.114	−1.604	−0.609	1.026	2.454
Item 25	2.817	−1.985	−1.430	−0.163	1.168	1.719
Item 26	3.179	−2.119	−1.526	−0.313	1.100	2.012
Item 27	3.465	−1.949	−1.507	−0.434	1.121	2.208
Item 28	4.390	−2.259	−1.568	−0.551	0.951	2.897
Item 29	3.277	−2.615	−1.759	−0.587	1.187	1.903
Item 30	3.577	−2.295	−1.512	−0.197	1.264	2.332
Item 31	2.940	−2.206	−1.401	−0.366	1.141	1.807
Item 32	2.708	−2.439	−1.628	−0.452	1.150	1.534
Item 33	2.602	−2.828	−1.848	−0.509	0.984	1.383

## Data Availability

The data that support the findings of this study are available from the corresponding author upon reasonable request. The data are not publicly available due to privacy or ethical restrictions.

## Supporting Information

Supporting file 1: The item characteristic curves of Item 1–Item 33 of the CCDNS-DRN.
