# Supplementary material for: Psychometric Evaluation of the Core Competencies in Disaster Nursing Scale for Disaster Rescue Nurses in Mainland China
Source: J Nurs Manag. 2025 Aug 10;2025:9957270. doi: 10.1155/jonm/9957270 (PMC12358227; doi:10.1155/jonm/9957270)
Supplement: Supporting Information — Additional supporting information can be found online in the Supporting Information section. [file 9957270.f1.pdf]

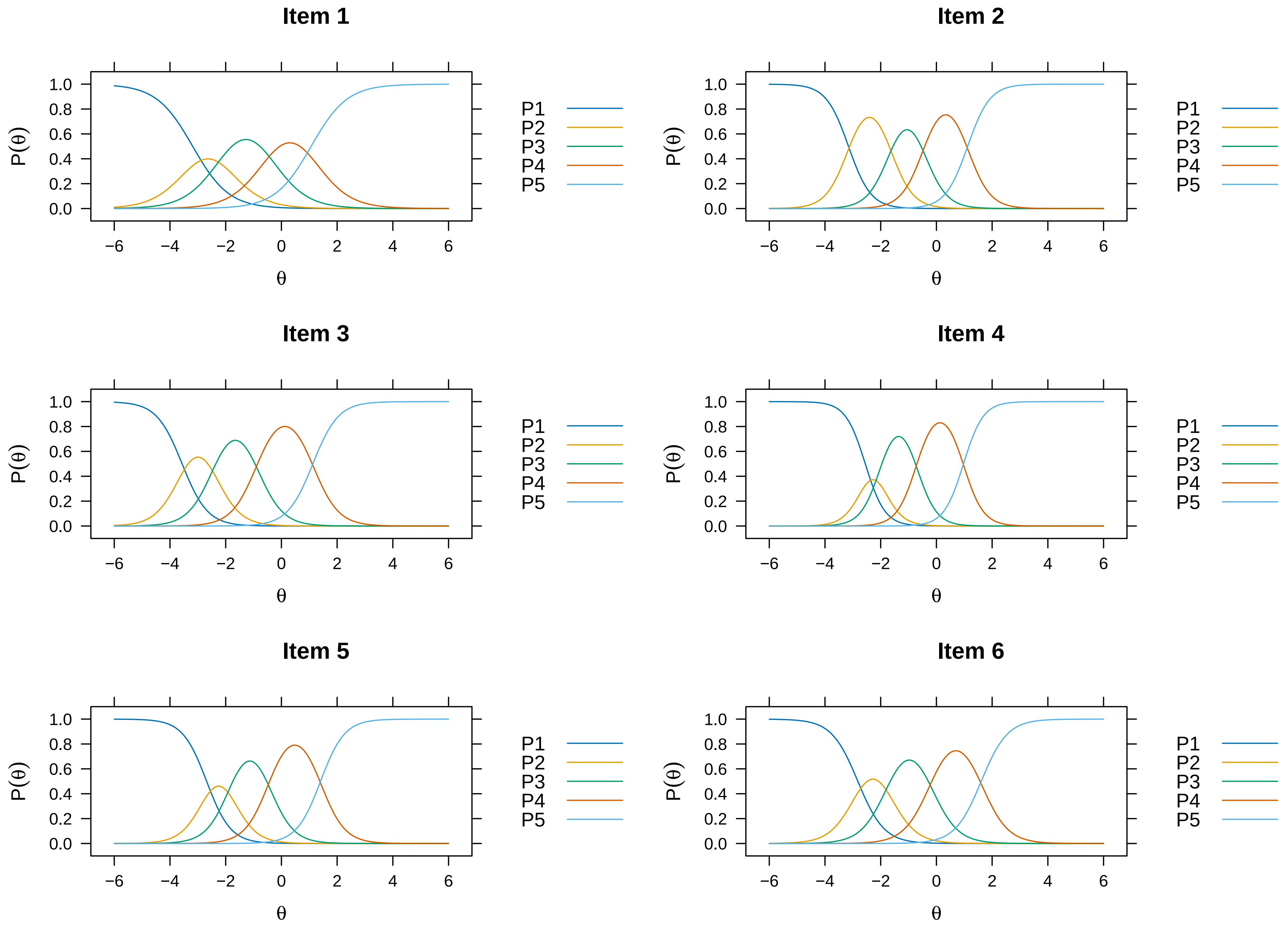
Item 1
Item 2
Item 3
Item 4
Item 5
Item 6
P(θ)
θ
−6
−4
−2
0
2
4
6
0.0
0.2
0.4
0.6
0.8
1.0
P1
P2
P3
P4
P5

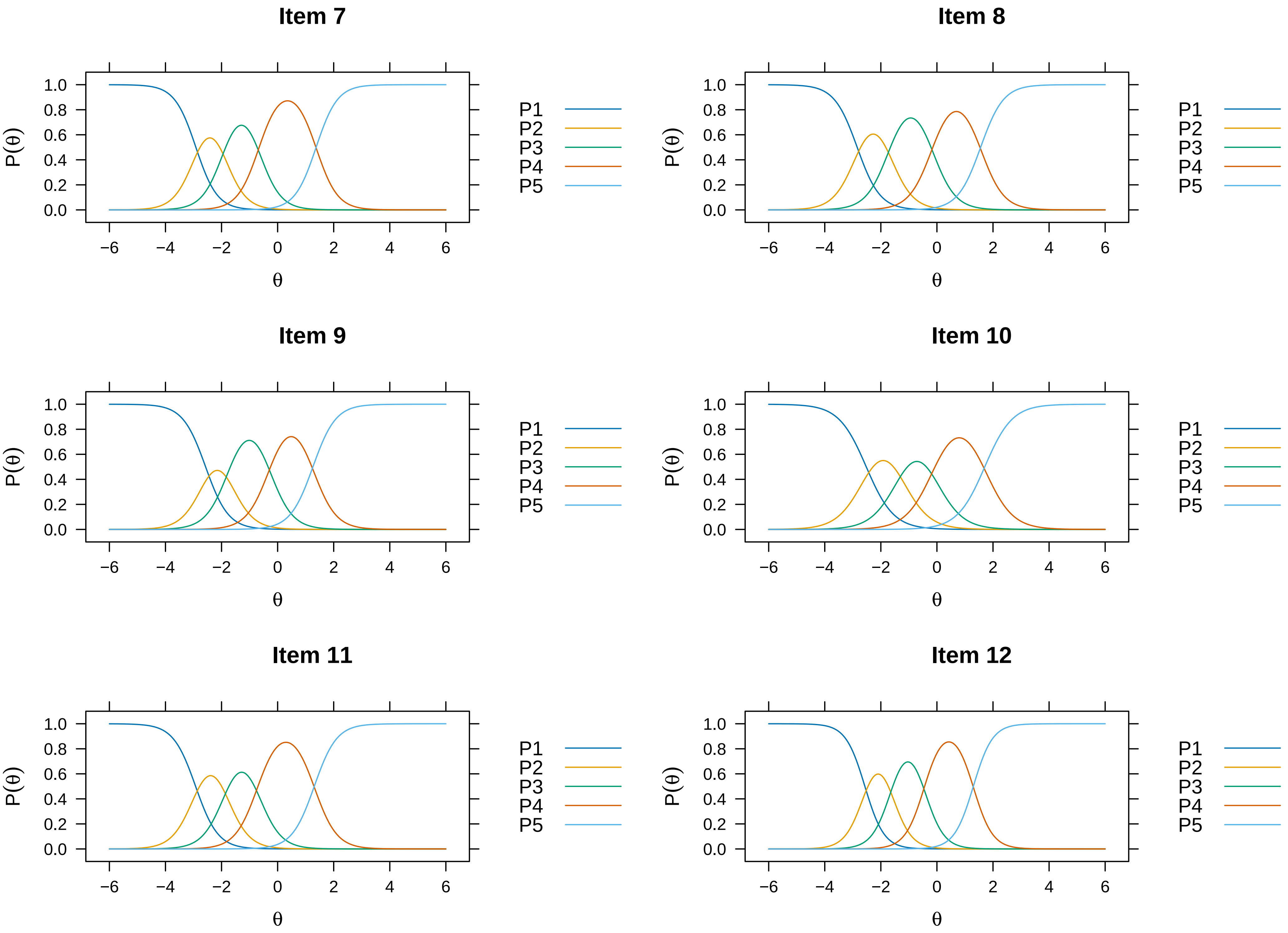

Item 7
Item 8
Item 9
Item 10
Item 11
Item 12
P(θ)
θ
1.0
0.8
0.6
0.4
0.2
0.0
−6 −4 −2 0 2 4 6
P1
P2
P3
P4
P5

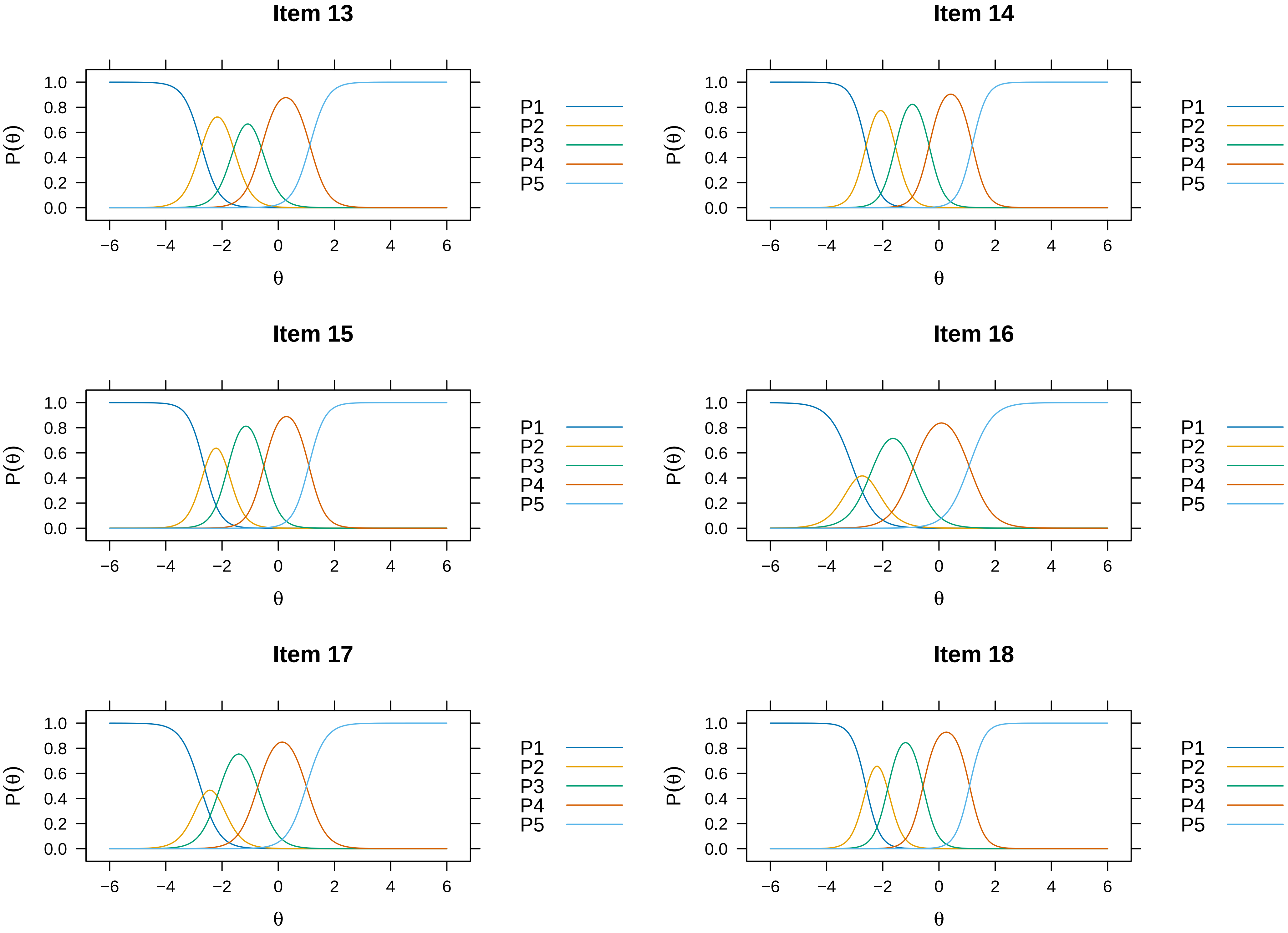
Item 13
Item 14
Item 15
Item 16
Item 17
Item 18
P(θ)
θ
P1
P2
P3
P4
P5
1.0
0.8
0.6
0.4
0.2
0.0
−6
−4
−2
0
2
4
6

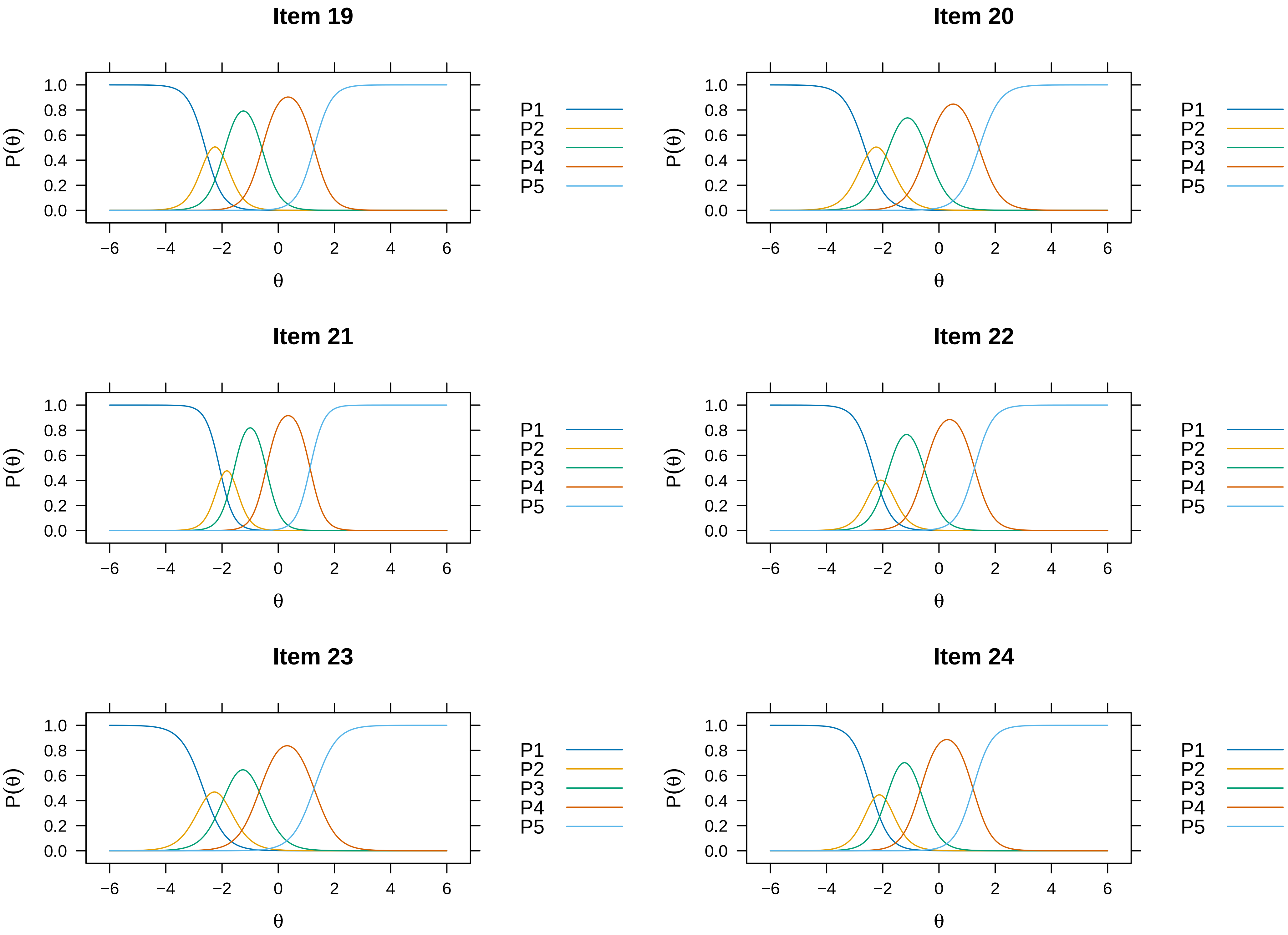

Item 19
Item 20
Item 21
Item 22
Item 23
Item 24
P(θ)
θ
1.0
0.8
0.6
0.4
0.2
0.0
−6
−4
−2
0
2
4
6
P1
P2
P3
P4
P5

Item 25

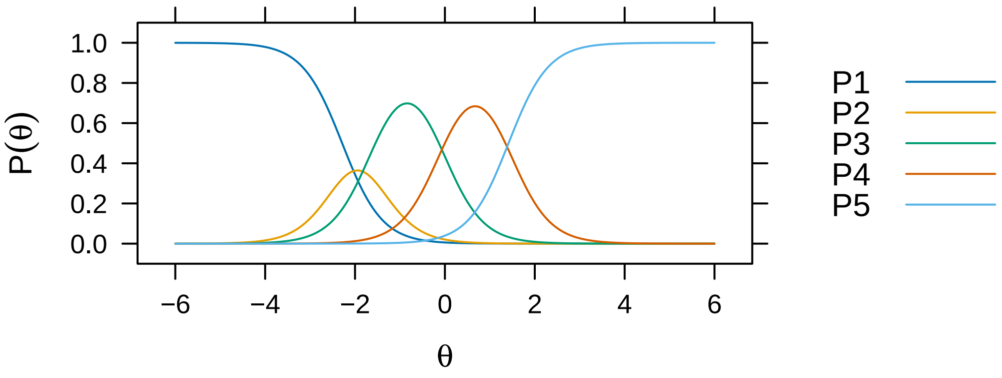

Item 26

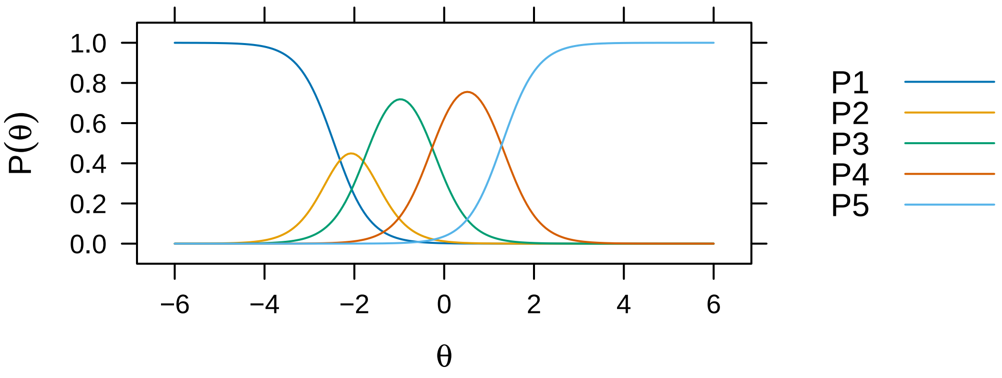

Item 27

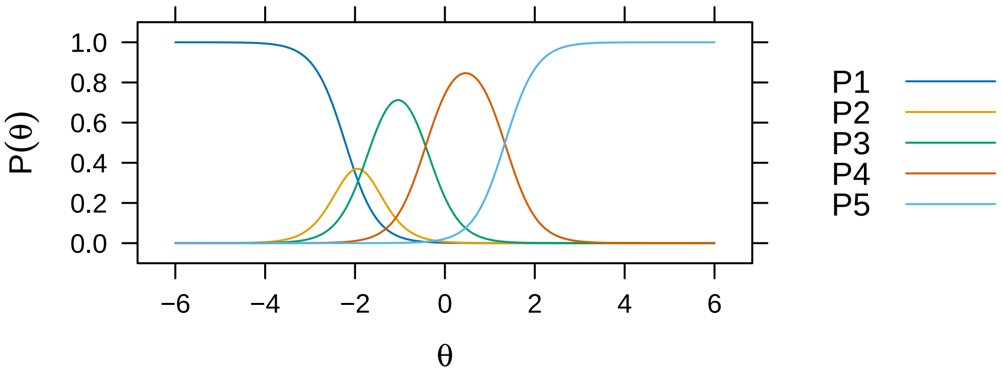

Item 28

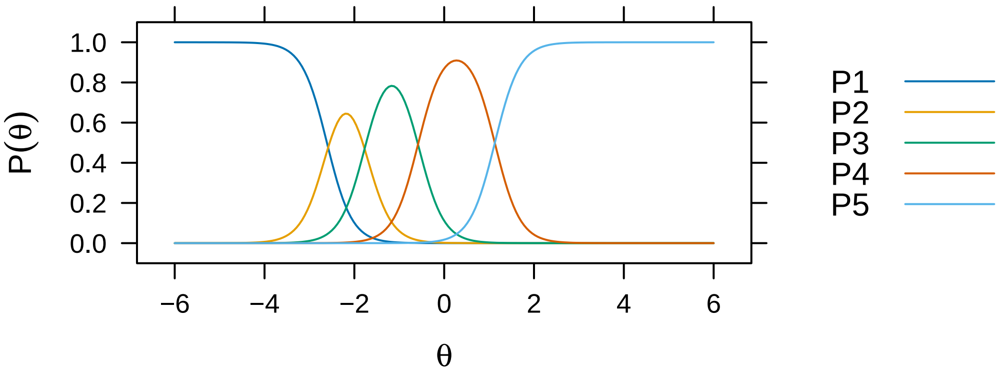

Item 29

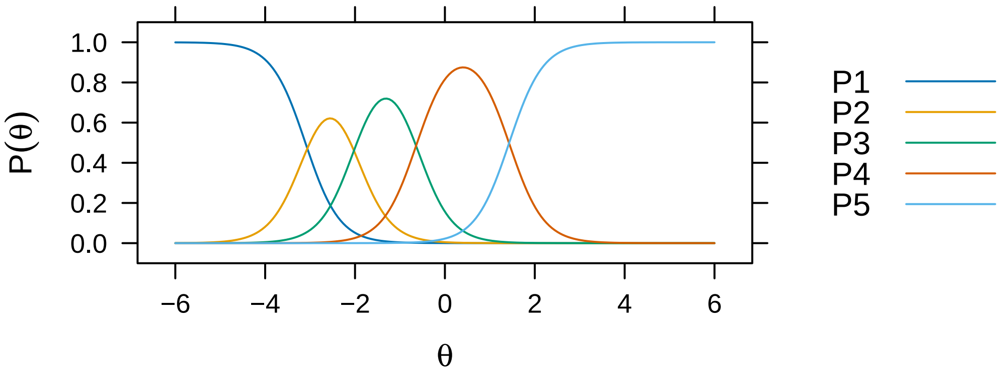

Item 30

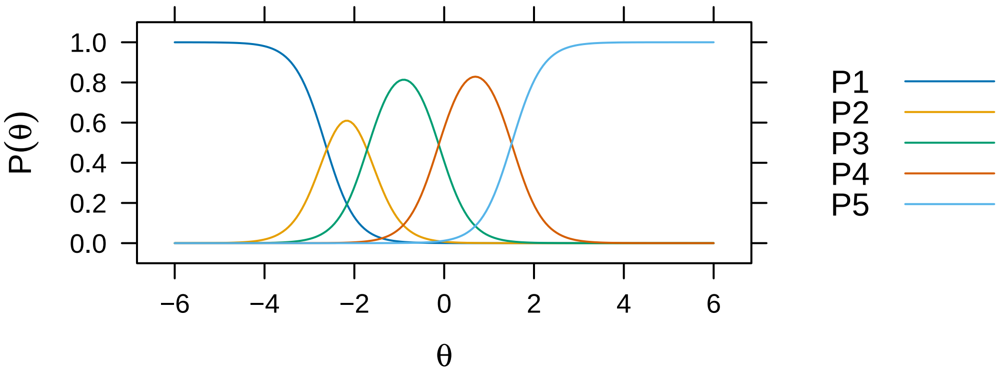

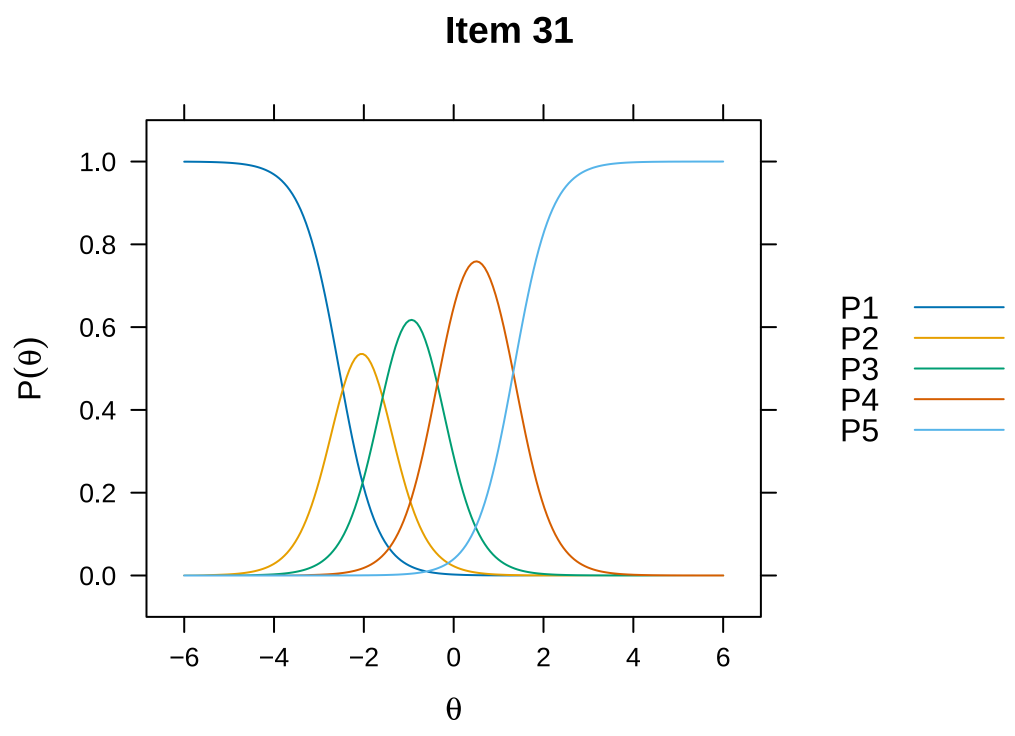

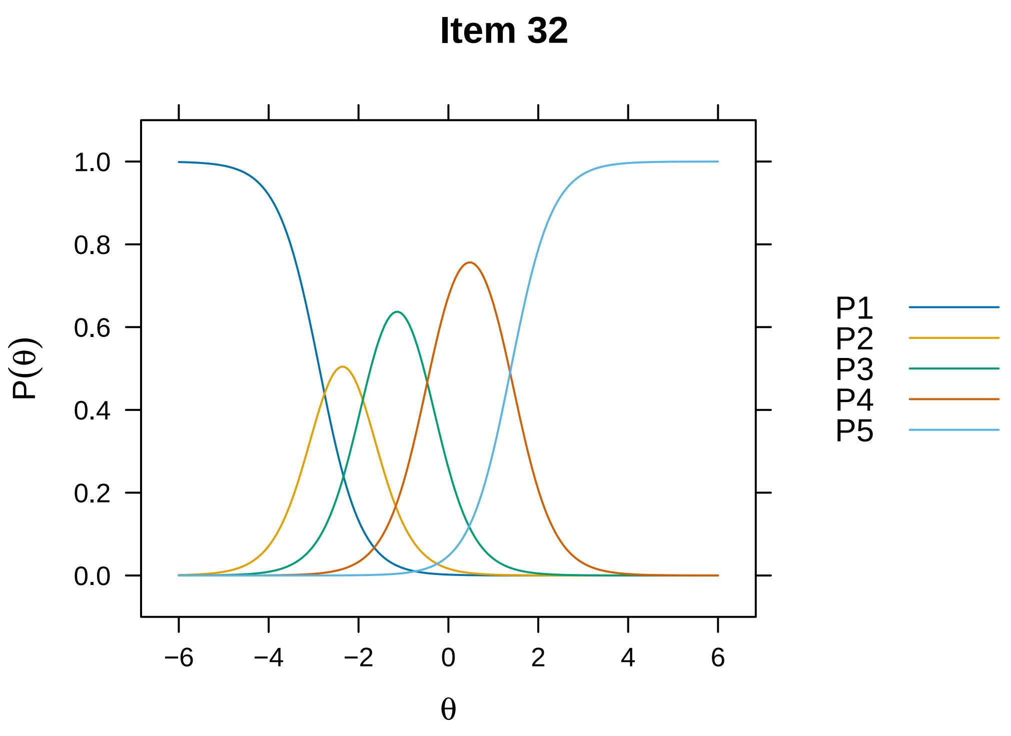

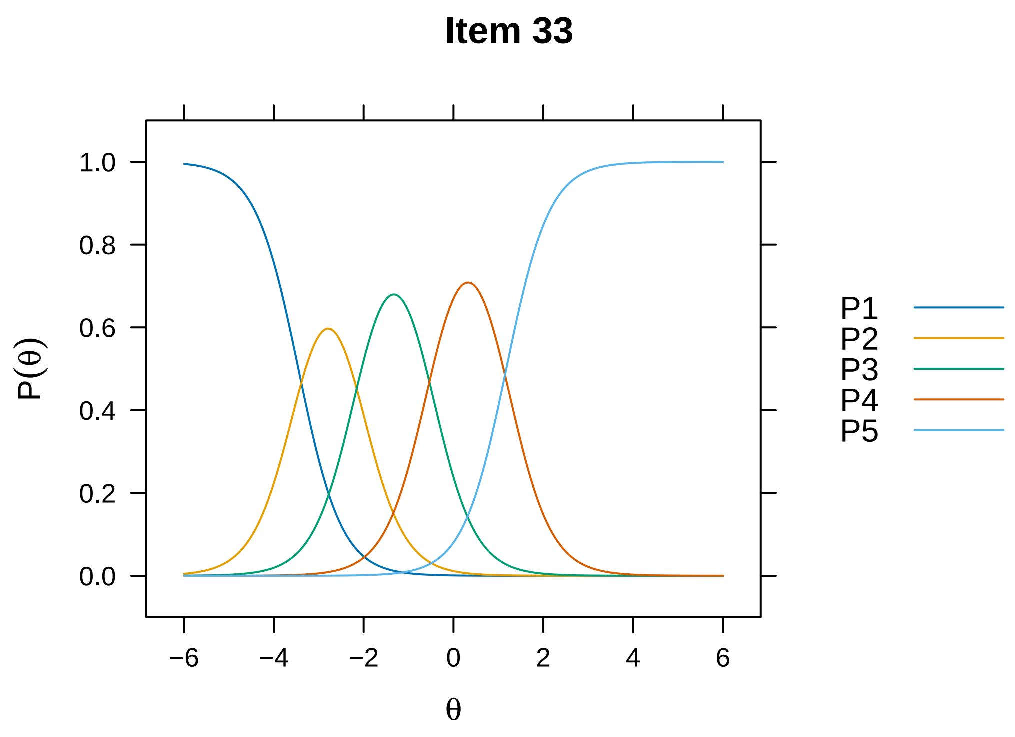

NOTE. The five curves in the figure correspond to the response probability of the subjects on the five options.
